# Dr. Eugene Wasdin

**Published:** 1911-12

**Authors:** 


Dr. Eugene Wasdin died in a sanitarium near Philadelphia, November 19, 1911, aged 53. Dr. Wasdin had long been a member of the U. S. Public Health and Marine Hospital Service. In 1901, he was stationed in Buffalo and assisted in the care of President McKinley.

